# External validation of the hospital frailty risk score among older adults receiving mechanical ventilation

**DOI:** 10.1038/s41598-022-18970-7

**Published:** 2022-08-26

**Authors:** Eric Sy, Sandy Kassir, Jonathan F. Mailman, Sarah L. Sy

**Affiliations:** 1 Department of Critical Care, Surgical Intensive Care Unit, Regina General Hospital, 1440 – 14 Avenue, Regina, SK S4P 0W5 Canada; 2 College of Medicine, University of Saskatchewan, Regina General Hospital, 1440 – 14 Avenue, Regina, SK S4P 0W5 Canada; 3 Research Department, Saskatchewan Health Authority, Wascana Rehabilitation Centre, 2180 – 23 Avenue, Regina, SK S4S 0A5 Canada; 4 Royal Jubilee Hospital, Vancouver Island Health Authority, Victoria, BC V8R 1J8 Canada; 5 Faculty of Pharmaceutical Sciences, University of British Columbia, Vancouver, BC V6T 1Z3 Canada; 6 Division of Geriatric Medicine, Department of Medicine, University of British Columbia, 7th Floor, GLDHCC, 2775 Laurel Street, Vancouver, BC V5Z 1M9 Canada

**Keywords:** Health care, Geriatrics

## Abstract

To externally validate the Hospital Frailty Risk Score (HFRS) in critically ill patients. We selected older adult (≥ 75 years old) hospitalizations receiving mechanical ventilation, using the Nationwide Readmissions Database (January 1, 2016-November 30, 2018). Frailty risk was subcategorized into low-risk (HFRS score < 5), intermediate-risk (score 5–15), and high-risk (score > 15). We evaluated the HFRS to predict in-hospital mortality, prolonged hospitalization, and 30-day readmissions, using multivariable logistic regression, adjusting for patient and hospital characteristics. Model performance was assessed using the c-statistic, Brier score, and calibration plots. Among 649,330 weighted hospitalizations, 9.5%, 68.3%, and 22.2% were subcategorized as low-, intermediate-, and high-risk for frailty, respectively. After adjustment, high-risk patient hospitalizations were associated with increased risks of prolonged hospitalization (adjusted odds ratio [aOR] 5.59 [95% confidence interval [CI] 5.24–5.97], c-statistic 0.694, Brier 0.216) and 30-day readmissions (aOR 1.20 [95% CI 1.13–1.27], c-statistic 0.595, Brier 0.162), compared to low-risk hospitalizations. Conversely, high-risk hospitalizations were inversely associated with in-hospital mortality (aOR 0.46 [95% CI 0.45–0.48], c-statistic 0.712, Brier 0.214). The HFRS was not successfully validated to predict in-hospital mortality in critically ill older adults. While it may predict other outcomes, its use should be avoided in the critically ill.

## Introduction

Frailty is increasingly being recognized as a risk factor for mortality, prolonged hospitalization, readmission, and poor quality of life after discharge in critically ill older adults^1–7^. Up to 24% of critically ill patients may be frail at baseline prior to admission^8^. Most studies have prospectively assessed frailty using the Canadian Study of Health and Aging Clinical Frailty Score (CFS)^4,8–10^. However, most frailty scores (i.e., CFS, Fried’s frailty phenotype, Edmonton Frail Scale) have limited use in administrative databases (i.e., Canadian Institute for Health Information Discharge Abstract Database, United States [US] Centers for Medicare & Medicaid Services databases), as these databases do not contain the information necessary to calculate these scores^10–13^. Hence, our understanding of frailty in critical illness has been limited to prospective studies.

Consequently, frailty scores for administrative databases have been developed, as interest in big data research increases. The electronic Frailty Index (eFI) was developed for use in primary care electronic records^14^. The modified Frailty Index (mFI) has been studied in Brazilian intensive care units (ICUs); however, it requires the measurement of functional capacity^15^. Recently, the Hospital Frailty Risk Score (HFRS) was derived using ridge regression and 109 *International Classification of Diseases, Tenth Revision Clinical Modification* (ICD-10-CM) codes, from a cohort of > 20,000 hospitalized older adults^16^. The HFRS has been validated to predict the risk of 30-day mortality, prolonged hospitalization, and 30-day emergency hospital readmissions in older hospitalized patients^16^, and it has since been validated in other hospitalized population databases^17–20^. However, its validity in critically ill patients has been questioned in a single center study^21^. Thus, there is a need to study the validity of the HFRS in large administrative databases of critically ill patients. The primary goal of this study was to externally validate the HFRS among a nationally representative US sample of older adults receiving mechanical ventilation.

## Methods

This study was reported using the transparent reporting of a multivariable prediction model for individual prognosis or diagnosis (TRIPOD) statement^22^. It was exempted by the Saskatchewan Health Authority Research Ethics Board (SHA-REB-20–77), as de-identified information was used, and it was performed in accordance with all institutional guidelines and regulations.

### Data source

We extracted information from the Nationwide Readmissions Database (NRD) from January 1, 2016, to November 30, 2018. The NRD is the largest all-payer US readmission database from the Healthcare Cost and Utilization Project (HCUP), and it includes hospitalizations of both insured and uninsured patients from 28 different state databases^23^. It samples > 15,000,000 unique hospitalizations annually, representing > 36,000,000 weighted hospitalizations, including general ward, intermediate care, and ICU patients^23^. The NRD accounts for 60% of the total US population and 59% of all hospitalizations, allowing for national estimates.

### Study population

We included all hospitalizations of older adults (≥ 75 years old) receiving mechanical ventilation, using a validated administrative definition in the ICD-10 procedure coding system (ICD-10-PCS) (Electronic Supplementary Material (ESM) eTable 1)^24^. We excluded patients who left against medical advice and hospitalizations with missing information for length of stay, time to next visit, and December admissions, as the NRD is unable to follow these patients beyond the calendar year. We also excluded hospitalizations of non-residents of the state, as the NRD does not have any linking state identifiers.

### Measurements

The covariates in the NRD included age, biological sex, hospital characteristics (teaching status, size), income quartile, primary insurance status (Medicare, Medicaid, private insurance, self-pay, or other) and Elixhauser-van Walraven comorbidity index score^25^. The ICD-10-CM and ICD-10-PCS codes were used to classify comorbidities (ESM eTable 1). We determined the primary reason for admission of the index hospitalization and readmission, using the first listed diagnosis (DX1) and aggregate groups of the Clinical Classifications Software Refined (CCSR) developed by HCUP (ESM eTable 2)^26^. Hospital costs were determined using total hospital charges multiplied by the all-payer cost-to-charge ratio, then inflation-adjusted to 2018 US dollars using the US Bureau of Labor Consumer Price Index for medical care^27,28^. Linked visits were identified through a linking variable.

### Frailty risk

Frailty risk was assessed using the HFRS developed by Gilbert et al. (ESM eTable 3)^16^. We classified patients as either low-risk (score < 5), intermediate-risk (score 5–15), or high-risk (score > 15) for frailty, based on the original HFRS study and subsequent validation studies^16–18^.

### Outcome(s)

We evaluated the performance of the HFRS to predict in-hospital mortality, as the primary outcome. The predictive performance of the HFRS for prolonged hospitalization and 30-day emergency hospital readmissions were evaluated as secondary outcomes. We only evaluated in-hospital all-cause mortality instead of 30-day mortality (inpatient or outpatient) because the NRD only records in-hospital deaths. We defined prolonged hospitalization as a hospital length of stay > 10 days and only evaluated 30-day emergency hospital readmissions, similar to Gilbert et al^16,17^.

### Statistical analysis

All statistical analyses were performed using Stata/MP 15.1 (College Station, Texas, US). A two-sided *p *value < 0.05 was considered statistically significant. We accounted for the complex sampling design of the NRD using sampling weights provided by HCUP^23^. Categorical variables were presented as unweighted numbers and weighted percentages. Continuous variables were presented as either means (standard deviation [SD]), or medians (interquartile range [IQR]), following testing for normality. Survey-specific Rao-Scott tests were used to compare nominal data. Survey-specific linear regression was used to compare continuous data, using the geometric means for non-normal data. Missing data were present in < 5% of all patient visits. As a result, a complete case analysis was performed for all analyses given the complex sampling design^29,30^.

We assessed the validity of the HFRS for predicting in-hospital mortality, prolonged hospitalization, and 30-day emergency hospital readmission, using unadjusted and adjusted logistic regression. For in-hospital mortality and prolonged hospitalization, we performed adjustment for age, biological sex, income quartile, insurance status, do-not-resuscitate status, admission diagnosis, hospital characteristics, and year. For 30-day emergency hospital readmissions, we performed adjustment for the same variables, also including hospital disposition. Model discrimination was assessed with the c-statistic and calibration with the Brier score^31,32^. Calibration plots additionally were constructed.

### Sensitivity analyses

We performed several sensitivity analyses to assess the robustness of our findings. First, we re-evaluated our findings using the HFRS as a continuous variable and using restricted cubic splines with five knots^33^. Next, we performed survey-specific Cox proportional hazards regression for in-hospital mortality and 30-day emergency hospital readmissions^34^. Subsequently, we derived 30-day in-hospital mortality, using hospitalization data from the NRD, and re-performed our primary analysis. We performed additional post hoc analyses, restricting the population to those who only received mechanical ventilation for greater than 24 h and restricting the population to only those who were admitted emergently. Additionally, subgroup analyses were performed for patients who received major operative procedures and those who did not. We also performed an additional sensitivity analysis adjusting for time receipt of mechanical ventilation. We then performed multiple imputation with chained equations for missing data using 10 imputations, and repeated the primary analysis with the imputed dataset^35^. Finally, as a post hoc analysis, we evaluated the total population of older adults in the NRD, independent of receiving mechanical ventilation, to determine whether our findings held for the entire older adult population.

### Ethics approval and consent to participate

This study was reviewed by the Saskatchewan Health Authority Research Ethics Board (REB-20–77) and was considered exempt under the TCPS2, with a waiver of consent.

### Preprint

A previous version of this manuscript was published as a preprint doi: 10.21203/rs.3.rs-1086390/v3, https://www.researchsquare.com/article/rs-1086390/v3

## Results

**Figure 1:**
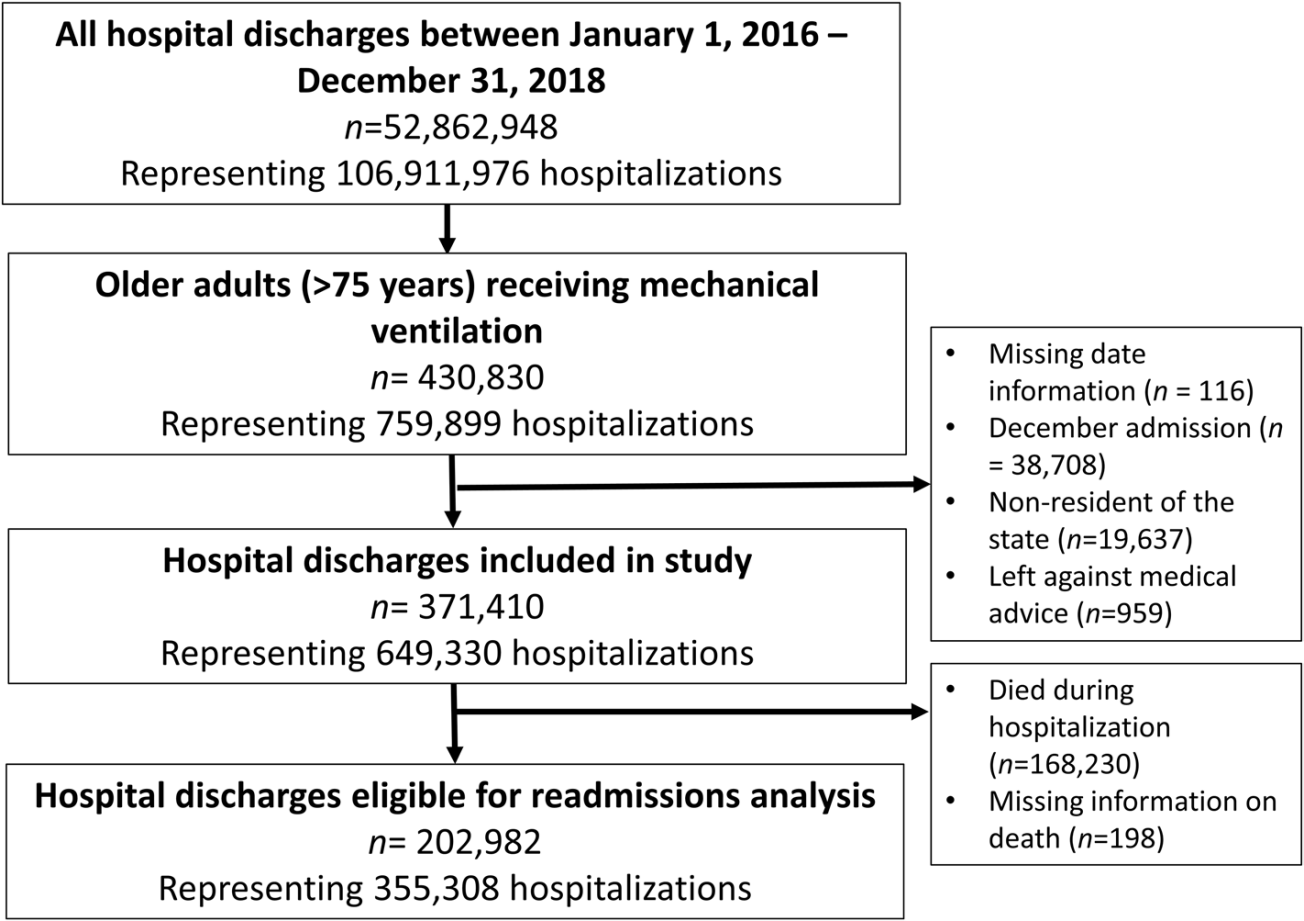
Flow chart of hospitalizations of older adults receiving invasive mechanical ventilation.

There were 371,410 hospitalizations of older adults receiving mechanical ventilation, representing 649,330 weighted hospitalizations (3.4% of all weighted hospitalizations in the database) (Fig. 1). A summary of baseline characteristics is described in Table 1 and ESM eTable 4. Missing data are described in ESM eTable 5. Of the hospitalizations, 50.0% had female patients, the median (IQR) age was 81 (78–86) years old, and the median (IQR) Elixhauser-van Walraven comorbidity index score was 18 (12–25). Infection-related diagnoses (30.5%) were the most common primary diagnoses. Many patients had primary or secondary diagnoses of severe sepsis (32.8%), shock (40.2%), and acute kidney injury (51.5%). Referral to palliative care occurred in approximately 26.8% of hospitalizations, with the high-risk for frailty group receiving the most referrals (p < 0.001).

**Table 1:** Characteristics of the population.

Characteristic^a^	Low-risk (HFRS < 5) *n* = 35,126	Intermediate-risk (HFRS 5–15) *n* = 253,711	High-risk (HFRS > 15) *n* = 82,573	Total population *n* = 371,410	*p *value^b^
Weighted number of hospitalizations	61,834	443,659	143,837	649,330	-
Age, median years (IQR)	81 (77–85)	81 (77–86)	82 (78–86)	81 (78–86)	< 0.001
**Patient characteristics**					
Female	18,250 (51.8)	125,932 (49.6)	41,809 (50.5)	185,991 (50.0)	< 0.001
Insurance					< 0.001
Medicare	32,185 (92.0)	232,555 (92.1)	75,020 (91.4)	339,760 (92.0)	
Medicaid	530 (1.3)	4,066 (1.4)	1,713 (1.8)	6,309 (1.5)	
Private	1,679 (4.6)	11,000 (4.2)	3,692 (4.3)	16,371 (4.2)	
Self-pay	153 (0.4)	940 (0.4)	257 (0.3)	1,350 (0.3)	
Other	549 (1.7)	4,883 (2.0)	1,824 (2.2)	7,256 (2.0)	
Household income quartile^c^					< 0.001
0-25th	9,915 (29.9)	68,798 (29.0)	22,772 (29.8)	101,485 (29.3)	
26-50th	9,442 (28.0)	66,073 (27.3)	20,702 (26)	96,217 (27.0)	
51-75th	8,393 (23.9)	61,505 (24.0)	20,077 (24.0)	89,975 (24.0)	
76-100th	6,946 (18.2)	54,416 (19.7)	18,201 (20.2)	79,563 (19.7)	
Elixhauser-van Walraven comorbidity index, median (IQR)	10 (5–16)	19 (12–25)	21 (15–27)	18 (12–25)	< 0.001
Hospital frailty risk score, median (IQR)	3.6 (2.3–4.3)	10.1 (7.9–12.3)	17.9 (16.3–20.3)	10.8 (7.7–14.5)	< 0.001
Elective admission	4,709 (14.0)	15,339 (6.5)	3,045 (4.1)	23,093 (6.7)	< 0.001
Major operative procedure performed	6,844 (19.7)	42,092 (16.8)	12,486 (15.4)	61,422 (16.7)	< 0.001
Time to mechanical ventilation, median days (IQR)	0 (0–1)	0 (0–2)	0 (0–3)	0 (0–2)	< 0.001
**Hospital characteristics**					
Hospital teaching status					< 0.001
Metropolitan non-teaching hospital	9,240 (24.6)	64,080 (23.5)	19,619 (21.9)	92,939 (23.2)	
Metropolitan teaching hospital	23,695 (67.4)	178,037 (70.6)	60,284 (73.7)	262,016 (71.0)	
Non-metropolitan hospital	2,191 (8.1)	11,594 (5.9)	2,670 (4.4)	16,455 (5.8)	
Hospital size					0.44
Small	4,328 (13.2)	31,850 (13.3)	9,872 (12.8)	46,050 (13.2)	
Medium	10,291 (28.1)	73,729 (28.0)	23,594 (27.6)	107,614 (28.0)	
Large	20,507 (58.8)	148,132 (58.7)	49,107 (59.6)	217,746 (58.9)	
**Outcomes**					
Length of stay, median days (IQR)	4 (1–8)	8 (4–15)	12 (7–21)	8 (4–15)	< 0.001
Long lengthy of stay (> 10 days)	6,086 (17.1)	100,002 (39.0)	47,895 (57.9)	153,983 (41.1)	< 0.001
In-hospital mortality	16,331 (46.4)	119,993 (47.3)	31,906 (38.6)	168,230 (45.3)	< 0.001
30-day emergency hospital readmission^d^	3,127 (16.4)	28,101 (20.6)	10,878 (20.9)	42,106 (20.3)	< 0.001

Abbreviations: interquartile range (IQR), standard deviation (SD).

^a^Expressed as unweighted number and weighted percentage (%) unless otherwise stated. Weighted percentages were calculated using complex survey methods in Stata and used the weighted number of hospitalizations.

^b^A *p *value < 0.05 considered statistically significant.

^c^As determined by the patient’s zip code.

^d^Among patient hospitalizations that survived their index admission (Unweighted total *n* = 18,775 for low-risk, *n* = 133,597 for intermediate-risk, *n* = 50,610 for high-risk, *n* = 202,982 total).

The median (IQR) HFRS was 10.8 (7.7–14.5) (ESM eFigure 1). Of all hospitalizations, 9.5% were classified as low-risk, 68.3% as intermediate-risk and 22.2% as high-risk.

### Prevalence of mortality, long hospital length of stay and 30-day hospital readmissions

In-hospital mortality occurred in 45.3% of all hospitalizations, and prolonged hospitalization occurred in 41.1% of all hospitalizations (Table 1). Of survivors, 20.3% were readmitted to hospital by 30 days. Among high-risk for frailty hospitalizations, they had an increased incidence of prolonged hospitalization and 30-day emergency hospital readmissions (all p < 0.001) compared to the low-risk for frailty group. However, they had a reduced incidence of in-hospital mortality compared to other frailty groups (p < 0.001).

### Assessment of model performance

**Figure 2:**
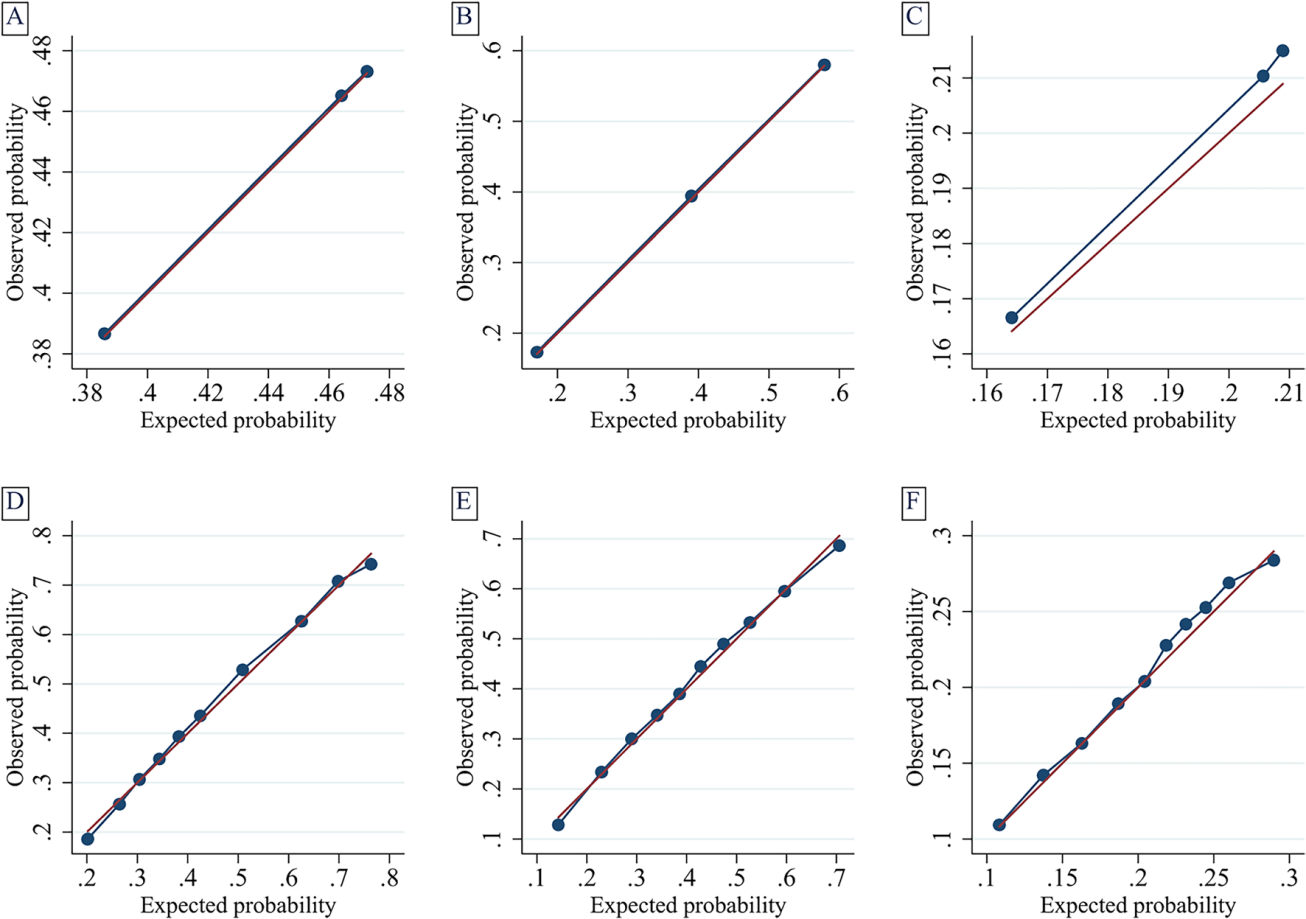
Calibration plots for logistic regression models. The red line refers to the reference slope or perfect calibration. The blue line refers to actual calibration slope of the model of interest. The dots refer to the observed frequency of events per tenth of predicted risk. (**A**) Unadjusted model for in-hospital mortality. (**B**) Unadjusted model for long hospital length of stay. (**C**) Unadjusted model for 30-day emergency hospital readmissions. (**D**) Adjusted model for in-hospital mortality. (**E**) Adjusted model for long hospital length of stay. (**F**) Adjusted model for 30-day emergency hospital readmissions.

Model performance was assessed for in-hospital mortality, prolonged hospitalization, and 30-day emergency hospital readmission (Table 2). In the unadjusted analysis, the intermediate- and high-risk groups were associated with reduced risk of in-hospital mortality, prolonged hospitalization, and increased risk of 30-day emergency hospital readmission. After adjustment, the intermediate- and high-risk for frailty groups were associated with reduced in-hospital mortality in this patient population (aOR 0.79 [95% CI 0.77–0.82] for intermediate-risk and aOR 0.46 [95% CI 0.45–0.48] for high-risk, c-statistic 0.712, Brier score 0.214), compared to the low risk for frailty group. Additionally, they were associated with prolonged hospitalization (aOR 2.61 [95% CI 2.46–2.78] for intermediate-risk and aOR 5.59 [95% CI 5.24–5.97] for high-risk, c-statistic 0.694, Brier score 0.216) and increased risk for 30-day emergency hospital readmission (aOR 1.18 [95% CI 1.12–1.24] for intermediate-risk and aOR 1.20 [95% CI 1.13–1.27] for high-risk, c-statistic 0.595, Brier score 0.162) after adjustment. Model calibration assessed using calibration plots (Fig. 2) visually demonstrate good calibration of the adjusted models.

**Table 2:** Model performance of HFRS subcategory and outcome in mechanically ventilated older adults.

Outcome	Unadjusted analysis	Adjusted analysis^a^
**In-hospital mortality**		
No. of unweighted hospitalizations in analysis	371,212	366,684
Low-risk HFRS, OR (95% CI)	1.00 (Reference)	1.00 (Reference)
Intermediate-risk HFRS, OR (95% CI)	1.03 (1.00–1.07)	0.79 (0.77–0.82)
High-risk HFRS, OR (95% CI)	0.73 (0.70–0.75)	0.46 (0.45–0.48)
C-statistic of the model	0.531 (0.529–0.533)	0.712 (0.710–0.714)
Brier score of the model	0.247	0.214
**Prolonged hospital length of stay (> 10 days)**		
No. of unweighted hospitalizations in analysis	371,410	366,881
Low-risk HFRS, OR (95% CI)	1.00 (Reference)	1.00 (Reference)
Intermediate-risk HFRS, OR (95% CI)	3.11 (2.93–3.29)	2.61 (2.46–2.78)
High-risk HFRS, OR (95% CI)	6.67 (6.27–7.10)	5.59 (5.24–5.97)
C-statistic of the model	0.606 (0.605–0.608)	0.694 (0.692–0.696)
Brier score of the model	0.221	0.216
**30-day emergency readmission**		
No. of unweighted hospitalizations in analysis^b^	202,928	200,006
Low-risk HFRS, OR (95% CI)	1.00 (Reference)	1.00 (Reference)
Intermediate-risk HFRS, OR (95% CI)	1.32 (1.26–1.38)	1.18 (1.12–1.24)
High-risk HFRS, OR (95% CI)	1.35 (1.27–1.42)	1.20 (1.13–1.27)
C-statistic of the model	0.513 (0.510–0.516)	0.595 (0.592–0.598)
Brier score of the model	0.164	0.162

Confidence interval (CI), hospital frailty risk score (HFRS), number (No.), odds ratio (OR).

^a^Adjusted for age (continuous variable), Elixhauser-van Walraven comorbidity index score (continuous variable), do-not-resuscitate status, biological sex, insurance status, income quartile, year of study, hospital teaching status, hospital size, and admission diagnosis category. 30-day emergency readmissions include additional adjustment for hospital disposition.

^b^Total number of patient hospitalizations in analysis who survived index hospital admission.

### Sensitivity analyses

**Figure 3:**
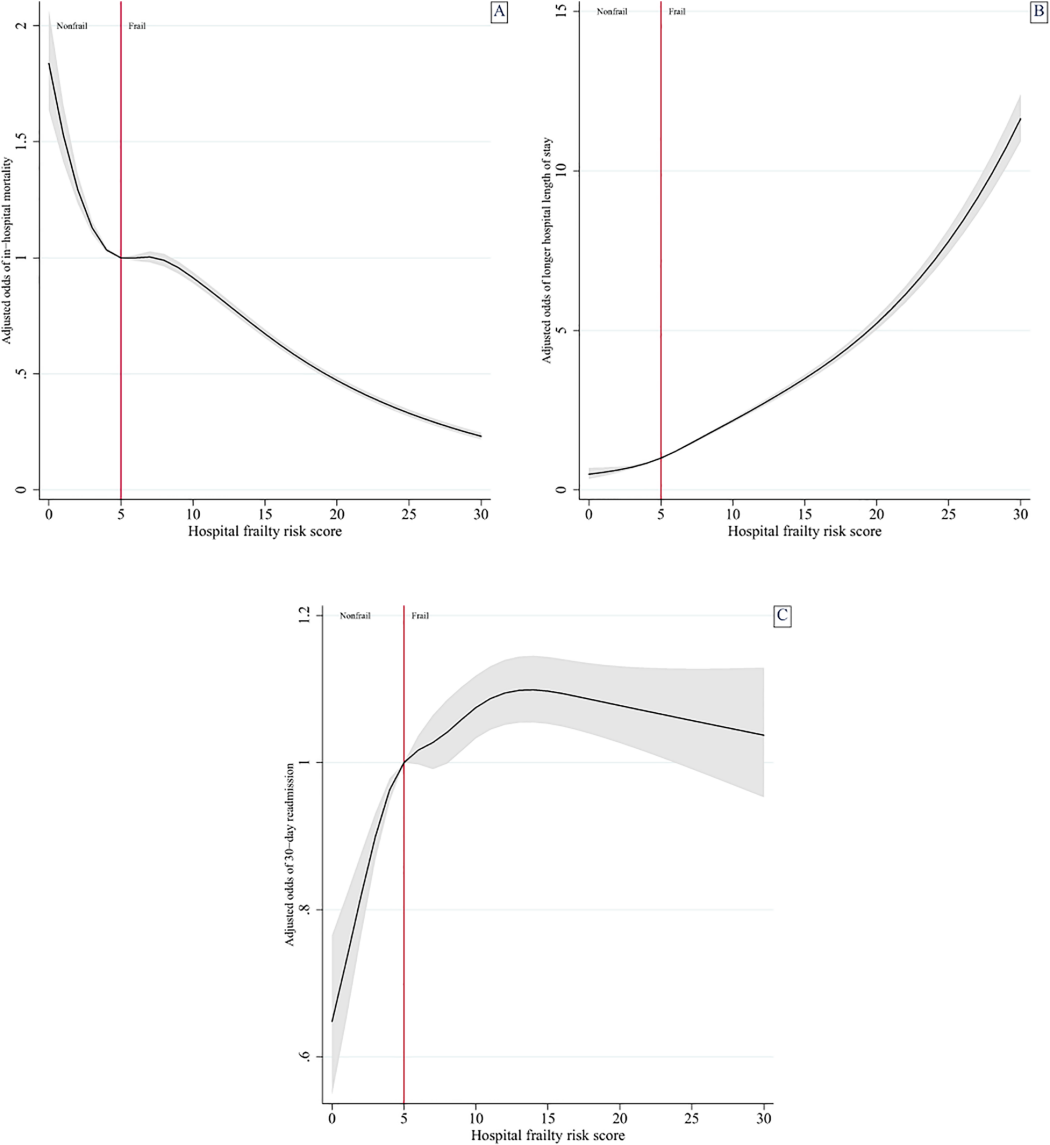
Association of the hospital frailty risk score (HFRS) with outcome of interest, using restricted cubic splines with five knots and an HFRS of 5, as the reference category. All models adjusted for age (continuous variable), Elixhauser-van Walraven comorbidity index score (continuous variable), do-not-resuscitate status, biological sex, insurance status, income quartile, year of study, hospital teaching status, hospital size, and admission diagnosis category. All spline graphs truncated at an HFRS of 30. (**A**) In-hospital mortality. (**B**) Longer hospital length of stay. (**C**) 30-day emergency hospital readmission, with additional adjustment for hospital disposition.

Detailed information on the sensitivity analyses is available in the ESM eResults and in eTable 6-e16. We performed several different analyses to evaluate the robustness of our analysis method, including re-analyzing our data using the HFRS as a continuous variable (ESM eTable 6) or using restricted cubic splines with five knots (ESM eTable 7, Fig. 3), performing Cox proportional hazards regression (ESM eTable 8), evaluating in-hospital 30-day mortality (ESM eTable 9), and performing multiple imputation with chained equations (ESM eTable 15). These additional analyses did not alter our overall findings.

## Discussion

In this study, the primary objective was to externally validate the HFRS to accurately predict in-hospital mortality in a large nationally representative cohort of older adults receiving mechanical ventilation. In its current form, the HFRS could not be successfully validated for use in this population. As expected, we found that patient hospitalizations receiving mechanical ventilation with intermediate- and high-risk for frailty, as categorized by the HFRS, were associated with increased risks of prolonged hospitalization and 30-day emergency hospital readmissions, compared to low-risk hospitalizations. Counterintuitively, they were inversely associated with in-hospital mortality when compared to the low-risk hospitalizations, suggestive of a potential spurious relationship. Regardless, the HFRS had only moderate discrimination and accuracy in predicting any of these outcomes. Using the HFRS as a continuous variable or with splines did not provide additional value over using the HFRS subcategories of low-, intermediate-, and high-risk. Our findings would suggest that clinicians and researchers should avoid using the HFRS when conducting big data research with administrative datasets of critically ill patients.

### Comparison with previous studies

Prior HFRS studies focused on validating it in general hospitalizations, including non-ICU and ICU patients^17–19,36–39^. Recently, there has been interest in externally validating the HFRS in ICU administrative databases, as interest in big data frailty research increases^21,40,41^. A German ICU study of 1,498 patients evaluated the HFRS to predict a combined endpoint of mortality and risk of readmission and found no association after adjustment for severity of illness^21^. In a large Wales population study, the HFRS had only moderate ability for predicting inpatient, 6-month, and 1-year mortality in hospital and ICU patients^41^. Conversely, a US study of 12,854 patients, using the single-center Medical Information Mart for Intensive Care (MIMIC-III) database, found that higher HFRS was associated with an increased risk of 28-day mortality^40,42^.

In our study, we found that critically ill older adult hospitalizations receiving mechanical ventilation were at high-risk of poor outcomes, including prolonged hospitalization (41%), 30-day in-hospital mortality (44%), in-hospital mortality (45%), and 30-day emergency hospital readmission (20%). Unsurprisingly, palliative care utilization was very high at 26.8%, with higher use in the high-risk frailty groups. The overall readmission rate was high in the patients of this study, suggestive of current difficulties in transitions in care for these patients and potential room for quality improvement.

Prior studies of critically ill patients have established that frailty is associated with increased risks of mortality^3,4^. Counterintuitively, we found that the HFRS was inversely associated with mortality in the NRD (i.e., lower HFRS was associated with the highest risks of in-hospital mortality). To ascertain this surprising and unnatural finding, we performed a post hoc analysis on the entire NRD population of older adults, independent of the receipt of mechanical ventilation, and found that the HFRS performed well on the *whole* population (i.e., higher HFRS was associated with the highest risks of in-hospital mortality in *all* older adults) (**ESM e**Table 13).

There may be some possible explanations for this unusual phenomenon, including selection biases and coding biases. In Gilbert et al.’s original study, they validated the HFRS in a general hospitalized population to predict in-hospital mortality^16^. In general, critically ill patients are at higher risk of death compared to a general hospitalized population, representing a surrogate endpoint. Therefore, by limiting our population to mechanically ventilated patients, selection bias may have been introduced, potentially altering the true association of the HFRS and mortality. Coding biases may also occur as critically ill patients who had prolonged hospitalizations and/or survived their hospitalization may appear to more “frail,” as they accrue more ICD-10-CM secondary diagnoses captured in their medical records. In the NRD, most of the hospitalizations of older adults receiving mechanical ventilation were in the intermediate-risk frailty group, and most hospitalizations in the high-risk group had significantly more ICD-10-CM codes captured compared to hospitalizations in the low-risk group. Finally, frail patients with higher severity of illness or those with treatment limitations may choose less invasive treatments, introducing further selection bias. We did adjust for do-not-resuscitate status; however, this may not fully capture all treatment limitations.

These biases and differences in the ICU patient population from the original development cohort could potentially explain why the HFRS had mixed performances for predicting in-hospital mortality in an ICU patient population, as seen in this study and others.

### Strengths and limitations

Our study had several strengths including the use of a large multicentre dataset, comprising close to 650,000 weighted hospitalizations. To our knowledge, our study represents one of the largest studies of critically ill patients examining the use of the HFRS, allowing for generalizability of our findings to critically ill older adults receiving mechanical ventilation. Unlike prior external validation studies in critically ill administrative databases, we evaluated the HFRS to predict prolonged hospitalization and 30-day emergency hospital readmissions. Additionally, we assessed both model discrimination and calibration, allowing for confidence in the results presented. Finally, our study performed several sensitivity analyses to verify our findings.

However, our study has limitations. As discussed previously, selection bias may have occurred in our selection of a mechanically ventilated population. The NRD was not designed specifically to flag admissions for critical care. Hence, the identification of critically ill patients was done through ICD-10 codes, specific to mechanical ventilation. Other codes, such as vasopressor use, are known to be significantly undercoded in administrative databases^43^. As the HFRS is derived from a composite of ICD-10 codes, coding practices and biases may affect the relative prevalence of admission comorbidities, diagnoses, and treatments. Some important codes to the determination of the HFRS, such as dementia in Alzheimer’s disease (F00) or care involving the use of rehabilitation procedures (Z50), were undercoded (ESM eTable 3). This is similarly seen in other databases including the Centers for Medicare & Medicaid Services and National Inpatient Sample databases^36,37^. Other databases of critically ill patients may perform differently, depending on their coding practices.

Additionally, the NRD does not have sufficient information to determine ICU severity of illness, such as the sequential organ failure assessment (SOFA) or Acute Physiologic Assessment and Chronic Health Evaluation II (APACHE II) scores. We are therefore unable to verify whether the HFRS would perform better after adequate adjustment for severity of illness; however, other studies would suggest that the HFRS does not perform well even after adjustment for severity of illness^21^. Likewise, the NRD does not capture detailed clinical information (i.e., patient weight, vasopressor dosing), and while it collects information on length of mechanical of ventilation, this information is often incomplete. Furthermore, it does not record out-of-hospital deaths, limiting our ability to only evaluate in-hospital mortality. Finally, we did not evaluate other scores as this was beyond the scope of our study. These limitations highlight the difficulty in applying the HFRS to datasets of critically ill patients and further support our caution on avoiding the use of the HFRS to predict these outcomes.

### Clinical implications, research implications, and future directions

Clinicians need to have accurate predictions of frailty and outcomes to identify patients who would benefit from early geriatric medicine referral, as well as to engage with patients and their families in shared decision-making, goals of care discussion, and end-of-life planning, and/or palliative care referral. Likewise, healthcare administrators need to have accurate estimates of the number of frail patients to plan and allocate healthcare services. Big data researchers need accurate scores to classify patients correctly.

While the HFRS may have utility in non-ICU databases, our study demonstrates its limitations in critically ill patients. The mFI is a promising alternative; however, it needs further development and validation for use with ICD-10-CM codes^15,44^. Perhaps the better solution for clinicians, researchers, and administrators would be to adapt and transform existing databases for frailty research. With other well-validated frailty scores such as the CFS, there is a compelling argument for its integration into routine clinical practice and inclusion in data capture. Future research should be performed to re-develop the HFRS or other scores with different weighting specifically for critically ill patients.

## Conclusion

In this large nationally representative external validation study of older adults receiving mechanical ventilation, the HFRS could not be validated to predict in-hospital mortality in this population. While the HFRS may predict prolonged hospitalization and 30-day emergency hospital readmissions, its use should be avoided in the critically ill. Further research with administrative databases is necessary to develop accurate, intuitive frailty scores in critically ill patients.

## Supplementary Information

Supplementary Information 1.

Supplementary Information 2.

## Data Availability

The Nationwide Readmissions Database is available through the Healthcare Cost and Utilization Project (https://www.hcup-us.ahrq.gov/nrdoverview.jsp).

## Abbreviations

aOR: Adjusted odds ratio
CI: Confidence Interval
ESM: Electronic supplementary material
HCUP: Healthcare cost and utilization project
HFRS: Hospital frailty risk score
ICD-10: *International classification of diseases, tenth revision*
ICU: Intensive care unit
NRD: Nationwide readmissions database
OR: Odds ratio
SD: Standard deviation
US: United States
