# Supplementary material for: External validation of the hospital frailty risk score among older adults receiving mechanical ventilation
Source: Sci Rep. 2022 Aug 26;12:14621. doi: 10.1038/s41598-022-18970-7 (PMC9418158; doi:10.1038/s41598-022-18970-7)
Supplement: Supplementary file 1 — Supplementary Information 1. [file 41598_2022_18970_MOESM1_ESM.docx]

Electronic Supplementary Material

### Contents

[Electronic Supplementary Material eResults 3](#_Toc107835827)

[eTable 1. ICD-10 diagnoses and procedures 4](#_Toc107835828)

[eTable 2. Aggregate Clinical Classifications Software Refined (CCSR) admission diagnosis categories 5](#_Toc107835829)

[eTable 3. Diagnoses included in the Hospital frailty risk score, the number of points associated with each diagnosis, and their prevalence in study cohort subcategories 6](#_Toc107835830)

[eTable 4. Additional characteristics of the population 10](#_Toc107835831)

[eTable 5. Key variables with missing data. 13](#_Toc107835832)

[eTable 6. Model performance of HFRS as a continuous variable and outcome in older adults receiving mechanical ventilation 14](#_Toc107835833)

[eTable 7. Model performance of HFRS using restricted cubic splines and outcome in older adults receiving mechanical ventilation 15](#_Toc107835834)

[eTable 8. Hospital frailty risk score subcategory and adverse outcomes in older adults receiving mechanical ventilation, using a Cox proportional hazards model 16](#_Toc107835835)

[eTable 9. Model performance of HFRS subcategory and in-hospital 30-day mortality in older adults receiving mechanical ventilation 17](#_Toc107835836)

[eTable 10. Model performance of HFRS subcategory and outcome in older adult mechanically ventilated patients who received mechanical ventilation for greater than 24 hours 18](#_Toc107835837)

[eTable 11. Model performance of HFRS subcategory and outcome in older mechanically ventilated adults who only were admitted to hospital for an emergency admission 19](#_Toc107835838)

[eTable 12. Model performance of HFRS subcategory and outcome in older mechanically ventilated adults who only were admitted to hospital and had a major operative procedure 20](#_Toc107835839)

[eTable 13. Model performance of HFRS subcategory and outcome in older mechanically ventilated adults who only were admitted to hospital and did not have a major operative procedure 21](#_Toc107835840)

[eTable 14. Model performance of HFRS subcategory and outcome in older mechanically ventilated adults who only were admitted to hospital, after additional adjustment for time to receipt of mechanical ventilation 22](#_Toc107835841)

[eTable 15. Model performance of HFRS subcategory and outcome in older adults receiving mechanical ventilation after multiple imputation with chained equations 23](#_Toc107835842)

[eTable 16. Model performance of HFRS subcategory and outcome in all hospitalized older adults 24](#_Toc107835843)

[eFigure 1. Distribution of hospital frailty risk index score 25](#_Toc107835844)

[Electronic Supplementary Material References 26](#_Toc107835845)

### Electronic Supplementary Material eResults

*Sensitivity analyses*

Several sensitivity analyses were performed to evaluate the robustness of our analysis method. First, we evaluated the Hospital Frailty Risk Score (HFRS) as a continuous variable (**Electronic Supplementary Material [ESM] eTable 6**) or using restricted cubic splines with five knots (**ESM eTable 7, Figure 3**), instead of using the HFRS subcategories of low-risk, intermediate-risk, and high-risk. We found that model performance was comparable to the HFRS subcategory models and yielded similar results.

Next, we evaluated our results using a Cox proportional hazards multiple variable regression model instead of using logistic regression (**ESM eTable 8**), and we found similar model performance. Afterwards, we evaluated in-hospital 30-day mortality as opposed to only in-hospital mortality (**ESM eTable 9**), to have more comparable analyses to those of Gilbert et al.^1,2^

We next analyzed a smaller subpopulation of mechanically ventilated patients to analyzing those who were ventilated greater than 24 hours only (**ESM eTable 10**) and analyzing a subpopulation of only emergency admissions (**ESM eTable 11**), to account for different coding biases that may occur for shorter periods of ventilation or elective admissions. These analyses again yielded similar results as our primary analysis.

Next, we analyzed a smaller subpopulation of mechanically ventilated patients who had undergone a major operative procedure (**ESM eTable 12**) as well as those who did not undergo a major operative procedure (**ESM eTable 13**). After multivariable analysis, the results were similar for model performance to our original model. We additionally performed a sensitivity analysis, accounting for time to receipt of mechanical ventilation by performing additional adjustment in our model variable model (**ESM eTable 14**), which had similar model performance as the original model.

Next, we performed multiple imputation with chained equations (**ESM eTable 15**), to account for the missing data (present in <5% of all hospitalizations), and again found relatively similar results to our primary analysis.

Finally, we evaluated the whole population of all older hospitalized adults in the Nationwide Readmissions Database (NRD), independent of receipt of mechanical ventilation (**ESM eTable 16**), to determine whether our findings held true to the whole population of older adults. In this instance, we found that unlike in the mechanically ventilated subpopulation, the HFRS performed very well, and it predicted in-hospital mortality, prolonged hospitalization, and 30-day hospital readmissions with a high degree of discrimination and accuracy.

### eTable 1. ICD-10 diagnoses and procedures

| **ICD-10 Diagnosis/Procedure** | **Associated ICD-10-CM/ICD-10-PCS codes^a^** |
| --- | --- |
| Acute kidney Injury (AKI) | N17 |
| Acute respiratory distress syndrome (ARDS) | J80 |
| Chronic kidney disease | N18 |
| Acute decompensated heart failure (ADHF) | I50.21, I50.23, I50.31, I50.33, I50.41, I50.43 |
| Dementia | F00, F01, F02, F03, G30.0, G30.1, G30.8, G30.9, G31.0, G31.09, G31.83 |
| Do not resuscitate status | Z66 |
| Renal replacement therapy (hemodialysis) | 5A1D00Z, 5A1D60Z, 5A1D70Z, 5A1D80Z, 5A1D90Z |
| Insertion of new gastrostomy tube | 0DH60UZ, 0DH63UZ, 0DH64UZ  **AND**  Absence of Z93.1 (prior gastrostomy) |
| Insertion of new tracheostomy | One of: 0B110F4, 0B113F4, or 0B114F4  **AND**  Absence of Z93.0 (prior tracheostomy) |
| Mechanical ventilation and/or intubation | 5A1935Z, 5A1945Z, 5A1955Z, 0BH17EZ |
| Prior gastrostomy tube | Z93.1 |
| Prior tracheostomy | Z93.0 |
| Referral to palliative care | Z51.5 |
| Severe sepsis with/without septic shock | R65.20, R65.21 |
| Shock | R57, R65.21, T78.2, T79.4, T81.10, T81.12 |
| Stroke | G46 |

Abbreviations: *International Classification of Diseases, Tenth Edition* (ICD-10), Clinical Modification (CM), Procedure Classification System (PCS)

^a^For 2016, the ICD-10-CM codes were searched up to 35 primary and secondary diagnosis codes, and the ICD-10-PCS were searched up to 15 primary and secondary procedure codes. For 2017 and 2018, the ICD-10-CM codes were searched up to 40 primary and secondary diagnosis codes, and the ICD-10-PCS were searched up to 25 primary and secondary procedure codes.

### eTable 2. Aggregate Clinical Classifications Software Refined (CCSR) admission diagnosis categories

| **Clinical Classifications Software Refined Body System – Admission diagnosis category^a^** | **Associated three-character abbreviation/codes** |
| --- | --- |
| Certain infectious and parasitic diseases | INF |
| Neoplasms | NEO |
| Diseases of the blood and blood-forming organs, and certain disorders involving the immune mechanism | BLD |
| Endocrine, nutritional, and metabolic diseases | END |
| Diseases of the nervous system | NVS |
| Diseases of the circulatory system | CIR |
| Diseases of the respiratory system | RSP |
| Diseases of the digestive system | DIG |
| Diseases of the genitourinary system | GEN |
| Injury, poisoning, and certain other consequences of external causes | INJ |
| Other | EAR, EXT, EYE, FAC, MAL, MBD, MUS, PNL, PRG, SKN, SYM |

^a^See reference for the Clinical Classifications Software Refined.^3^ Only the primary diagnosis (i.e., DX1) was used for the CCSR diagnoses. The CCSR was developed to provide clinically meaningful categories of diagnoses, based on ICD-10-CM codes.

### eTable 3. Diagnoses included in the Hospital frailty risk score, the number of points associated with each diagnosis, and their prevalence in study cohort subcategories

| **Description of ICD-10 diagnosis^a^** | **Associated ICD-10 code^b^** | **Number of HFRS points associated with diagnosis^c^** | **Prevalence of diagnosis in the low-risk (score <5) for frailty subcategory (%)^d^** | **Prevalence of diagnosis in the intermediate-risk (score 5-15) for frailty subcategory (%)^d^** | **Prevalence of diagnosis in the high-risk (score >15) for frailty subcategory (%)^d^** |
| --- | --- | --- | --- | --- | --- |
| 1. Dementia in Alzheimer's disease | F00 | 7.1 | 0% | 0% | 0% |
| 1. Hemiplegia | G81 | 4.4 | 0.1% | 2.9% | 13.2% |
| 1. Alzheimer's disease | G30 | 4 | 0.2% | 2.8% | 10.4% |
| 1. Sequelae of cerebrovascular disease | I69 | 3.7 | 0.2% | 3.5% | 16.6% |
| 1. Other symptoms and signs involving the nervous and musculoskeletal systems | R29 | 3.6 | 0.1% | 2.2% | 11.4% |
| 1. Other disorders of urinary system | N39 | 3.2 | 0.6% | 13.4% | 50.3% |
| 1. Delirium, not induced by alcohol and other psychoactive substances | F05 | 3.2 | 0.1% | 2.2% | 8.2% |
| 1. Unspecified fall | W19 | 3.2 | 0.2% | 2.1% | 7.4% |
| 1. Superficial injury of head | S00 | 3.2 | 0.03% | 0.5% | 2.5% |
| 1. Unspecified hematuria | R31 | 3 | 0.2% | 1.8% | 6.1% |
| 1. Other bacterial agents as the cause of diseases classified elsewhere | B96 | 2.9 | 0.1% | 4.2% | 23.8% |
| 1. Other symptoms and signs involving cognitive functions and awareness | R41 | 2.7 | 0.5% | 2.8% | 8.5% |
| 1. Abnormalities of gait and mobility | R26 | 2.6 | 0.1% | 0.8% | 3.4% |
| 1. Other cerebrovascular diseases | I67 | 2.6 | 0.2% | 0.8% | 2.8% |
| 1. Convulsions, not elsewhere classified | R56 | 2.6 | 0.5% | 2.3% | 5.8% |
| 1. Somnolence, stupor, and coma | R40 | 2.5 | 4.3% | 8.4% | 15.9% |
| 1. Complications of genitourinary prosthetic device, implants, and grafts | T83 | 2.4 | 0.03% | 0.7% | 4.8% |
| 1. Intracranial injury | S06 | 2.4 | 0.8% | 3.1% | 6.9% |
| 1. Fracture of shoulder and upper arm | S42 | 2.3 | 0.1% | 0.5% | 1.6% |
| 1. Other disorders of fluid, electrolyte, and acid-base balance | E87 | 2.3 | 14.9% | 67.1% | 83.1% |
| 1. Other joint disorders, not elsewhere classified | M25 | 2.3 | 0.1% | 0.7% | 1.9% |
| 1. Volume depletion | E86 | 2.3 | 0.8% | 10.2% | 27.1% |
| 1. Senility | R54 | 2.2 | 0.1% | 0.4% | 0.8% |
| 1. Care involving use of rehabilitation procedures | Z50 | 2.1 | 0% | 0% | 0% |
| 1. Unspecified dementia | F03 | 2.1 | 2.9% | 11.7% | 25.1% |
| 1. Other fall on same level | W18 | 2.1 | 0.4% | 2.1% | 4.7% |
| 1. Problems related to medical facilities and other health care | Z75 | 2 | 0.01% | 0.1% | 0.3% |
| 1. Vascular dementia | F01 | 2 | 0.1% | 0.7% | 3.0% |
| 1. Superficial injury of lower leg | S80 | 2 | 0.04% | 0.2% | 0.9% |
| 1. Cellulitis | L03 | 2 | 0.3% | 2.6% | 5.2% |
| 1. Blindness and low vision | H54 | 1.9 | 0.3% | 1.0% | 2.4% |
| 1. Deficiency of other B group vitamins | E53 | 1.9 | 0.1% | 0.7% | 1.7% |
| 1. Problems related to social environment | Z60 | 1.8 | 0.1% | 0.1% | 0.3% |
| 1. Parkinson's disease | G20 | 1.8 | 0.8% | 2.6% | 5.6% |
| 1. Syncope and collapse | R55 | 1.8 | 0.5% | 0.9% | 1.6% |
| 1. Fracture of rib(s), sternum and thoracic spine | S22 | 1.8 | 0.6% | 2.5% | 4.1% |
| 1. Other functional intestinal disorders | K59 | 1.8 | 1.2% | 4.7% | 9.9% |
| 1. Acute renal failure | N17 | 1.8 | 11.6% | 52.4% | 65.9% |
| 1. Decubitus ulcer | L89 | 1.7 | 0.6% | 6.9% | 19.9% |
| 1. Carrier of infectious disease | Z22 | 1.7 | 0.1% | 0.5% | 1.4% |
| 1. Streptococcus and staphylococcus as the cause of diseases classified elsewhere | B95 | 1.7 | 0.3% | 2.1% | 7.8% |
| 1. Ulcer of lower limb, not elsewhere classified | L97 | 1.6 | 0.3% | 1.6% | 3.1% |
| 1. Other symptoms and signs involving general sensations and perceptions | R44 | 1.6 | 0.1% | 0.3% | 0.7% |
| 1. Duodenal ulcer | K26 | 1.6 | 0.5% | 1.4% | 2.1% |
| 1. Hypotension | I95 | 1.6 | 6.3% | 14.1% | 19.7% |
| 1. Unspecified renal failure | N19 | 1.6 | 0.2% | 0.2% | 0.2% |
| 1. Other septicemia | A41 | 1.6 | 8.4% | 40.8% | 58.7% |
| 1. Personal history of other disease and conditions | Z87 | 1.5 | 16.9% | 25.9% | 29.1% |
| 1. Respiratory failure, not elsewhere classified | J96 | 1.5 | 58.2% | 85.7% | 91.8% |
| 1. Exposure to unspecific factor | X59 | 1.5 | 0% | 0% | 0% |
| 1. Other arthrosis | M19 | 1.5 | 4.0% | 8.3% | 12.3% |
| 1. Epilepsy | G40 | 1.4 | 1.5% | 5.1% | 12.0% |
| 1. Osteoporosis without pathological fracture | M81 | 1.4 | 1.8% | 4.1% | 6.5% |
| 1. Fracture of femur | S72 | 1.4 | 0.5% | 1.8% | 3.4% |
| 1. Fracture of lumbar spine and pelvis | S32 | 1.4 | 0.2% | 0.8% | 1.8% |
| 1. Other disorders of pancreatic internal secretion | E16 | 1.4 | 0.2% | 1.3% | 2.4% |
| 1. Abnormal results of function studies | R94 | 1.4 | 0.2% | 0.5% | 0.9% |
| 1. Chronic renal failure | N18 | 1.4 | 13.6% | 35.9% | 43.3% |
| 1. Retention of urine | R33 | 1.3 | 1.1% | 3.6% | 8.7% |
| 1. Unknown and unspecified causes of morbidity | R69 | 1.3 | 0.003% | 0.003% | 0.003% |
| 1. Other disorders of kidney and ureters, not elsewhere classified | N28 | 1.3 | 1.0% | 2.0% | 3.4% |
| 1. Unspecified urinary incontinence | R32 | 1.2 | 0.3% | 0.9% | 2.8% |
| 1. Other degenerative disease of the nervous system, not elsewhere classified | G31 | 1.2 | 0.3% | 0.8% | 1.8% |
| 1. Nosocomial condition | Y95 | 1.2 | 0.6% | 4.1% | 7.5% |
| 1. Other and unspecified injuries of head | S09 | 1.2 | 0.1% | 0.2% | 0.5% |
| 1. Symptoms and signs involving emotional state | R45 | 1.2 | 0.3% | 0.9% | 2.3% |
| 1. Transient cerebral ischemic attacks and related syndromes | G45 | 1.2 | 0.2% | 0.3% | 0.5% |
| 1. Problems related to care-provider dependency | Z74 | 1.1 | 0.2% | 1.3% | 4.8% |
| 1. Other soft tissue disorder, not elsewhere classified | M79 | 1.1 | 0.7% | 1.4% | 2.2% |
| 1. Fall involving bed | W06 | 1.1 | 0.1% | 0.4% | 0.9% |
| 1. Open wound of head | S01 | 1.1 | 0.3% | 1.1% | 2.2% |
| 1. Other bacterial intestinal infections | A04 | 1.1 | 0.3% | 3.0% | 6.3% |
| 1. Diarrhea and gastroenteritis of presumed infectious origin | A09 | 1.1 | 0.01% | 0.1% | 0.2% |
| 1. Pneumonia, organism unspecified | J18 | 1.1 | 9.4% | 22.3% | 25.3% |
| 1. Pneumonitis due to solids and liquids | J69 | 1 | 6.0% | 19.6% | 32.5% |
| 1. Speech disturbances, not elsewhere classified | R47 | 1 | 0.7% | 2.8% | 10.3% |
| 1. Vitamin D deficiency | E55 | 1 | 0.5% | 1.5% | 2.7% |
| 1. Artificial opening status | Z93 | 1 | 1.6% | 4.9% | 11.0% |
| 1. Gangrene, not elsewhere classified | R02 | 1 | 0% | 0% | 0% |
| 1. Symptoms and signs concerning food and fluid intake | R63 | 0.9 | 0.6% | 1.2% | 2.0% |
| 1. Other hearing loss | H91 | 0.9 | 1.0% | 2.3% | 3.6% |
| 1. Fall on and from stairs and steps | W10 | 0.9 | 0.2% | 0.6% | 0.8% |
| 1. Fall on same level from slipping, tripping, and stumbling | W01 | 0.9 | 0.6% | 1.3% | 2.1% |
| 1. Thyrotoxicosis (hyperthyroidism) | E05 | 0.9 | 0.2% | 0.5% | 0.8% |
| 1. Scoliosis | M41 | 0.9 | 0.3% | 0.6% | 0.8% |
| 1. Dysphagia | R13 | 0.8 | 3.1% | 10.7% | 26.6% |
| 1. Dependence on enabling machines and devices | Z99 | 0.8 | 7.8% | 16.9% | 19.5% |
| 1. Agent resistant to penicillin and related antibiotics | U80 | 0.8 | 0% | 0% | 0% |
| 1. Osteoporosis with pathological fracture | M80 | 0.8 | 0.1% | 0.2% | 0.4% |
| 1. Other diseases of digestive system | K92 | 0.8 | 3.0% | 6.3% | 8.0% |
| 1. Cerebral infarction | I63 | 0.8 | 2.2% | 5.4% | 14.8% |
| 1. Calculus of kidney and ureter | N20 | 0.7 | 0.2% | 0.5% | 1.2% |
| 1. Mental and behavioural disorders due to use of alcohol | F10 | 0.7 | 1.2% | 2.3% | 2.9% |
| 1. Other medical procedures as the cause of abnormal reaction of the patient, or of later complication, without mention of misadventure at the time of the procedure | Y84 | 0.7 | 1.9% | 3.1% | 5.2% |
| 1. Abnormalities of heart beat | R00 | 0.7 | 4.9% | 6.9% | 9.2% |
| 1. Unspecified acute lower respiratory infection | J22 | 0.7 | 0.01% | 0.04% | 0.04% |
| 1. Problems related to life-management difficulty | Z73 | 0.6 | 0.01% | 0.004% | 0.01% |
| 1. Other abnormal findings of blood chemistry | R79 | 0.6 | 1.6% | 3.0% | 3.9% |
| 1. Personal history of risk factor, not elsewhere classified | Z91 | 0.5 | 3.8% | 5.7% | 8.1% |
| 1. Open wound of forearm | S51 | 0.5 | 0.04% | 0.2% | 0.4% |
| 1. Depressive episode | R32 | 0.5 | 5.2% | 8.3% | 11.8% |
| 1. Other spondylopathies | M48 | 0.5 | 1.2% | 2.0% | 2.9% |
| 1. Disorders of mineral metabolism | E83 | 0.4 | 4.1% | 13.4% | 19.0% |
| 1. Polyarthrosis | M15 | 0.4 | 0.2% | 0.3% | 0.3% |
| 1. Other anemias | D64 | 0.4 | 8.9% | 17.3% | 23.8% |
| 1. Other local infections of skin and subcutaneous tissue | L08 | 0.4 | 0.03% | 0.1% | 0.2% |
| 1. Nausea and vomiting | R11 | 0.3 | 0.8% | 0.9% | 1.2% |
| 1. Other noninfective gastroenteritis and colitis | K52 | 0.3 | 0.5% | 1.3% | 1.5% |
| 1. Fever of unknown origin | R50 | 0.1 | 0.7% | 1.0% | 1.2% |

Abbreviations: Hospital frailty risk score (HFRS), *International Classification of Diseases, Tenth Edition* (ICD-10)

^a^The components of the HFRS are derived from 109 individual different ICD-10 code diagnoses, based on Gilbert et al study,^1^ each with different weighting to determine the final HFRS score.

^b^For 2016, the ICD-10-CM codes were searched up to 35 primary and secondary diagnosis codes, and the ICD-10-PCS were searched up to 15 primary and secondary procedure codes. For 2017 and 2018, the ICD-10-CM codes were searched up to 40 primary and secondary diagnosis codes, and the ICD-10-PCS were searched up to 25 primary and secondary procedure codes.

^c^The number of points assigned to each ICD-10 code if present, based on Gilbert et al study.^1^ The HFRS for a hospitalization is calculated from the sum of the points of 109 different ICD-10 code diagnoses. For hospitalizations without the associated ICD-10 code, they would receive 0 points for each respective category.

^d^Weighted percentage of the number of hospitalizations within risk category with above comorbidity.

### eTable 4. Additional characteristics of the population

| **Characteristic^a^** | **Low-risk (HFRS <5)**  *n*=35,126 | **Intermediate-risk (HFRS 5-15)**  *n*=253,711 | **High-risk (HFRS >15)**  *n*=82,573 | **Total population**  *n*=371,410 | **p-value^b^** |
| --- | --- | --- | --- | --- | --- |
| Weighted number of hospitalizations | 61,834 | 443,659 | 143,837 | 649,330 | - |
| Do not resuscitate status | 10,011 (28.3) | 90,727 (36.1) | 30,895 (38.3) | 131,633 (35.8) | <0.001 |
| Total number of primary and secondary diagnoses codes, median (IQR) | 14 (10-18) | 20 (17-25) | 26 (23-31) | 21 (17-26) | <0.001 |
| **Admission diagnoses/comorbidities** |  |  |  |  |  |
| Acute stroke | * | 96 (0.03) | 113 (0.1) | * |  |
| ADHF | 6,005 (17.0) | 60,390 (23.9) | 15,675 (19.1) | 82,070 (22.2) | <0.001 |
| AKI | 4,131 (11.6) | 133,410 (52.4) | 54,487 (65.9) | 192,028 (51.5) | <0.001 |
| ARDS | 301 (0.8) | 3,243 (1.3) | 823 (1.0) | 4,367 (1.2) | <0.001 |
| CKD | 4,758 (13.6) | 90,293 (35.9) | 35,282 (43.3) | 130,333 (35.4) | <0.001 |
| Dementia | 1,233 (3.5) | 41,917 (16.2) | 32,863 (39.1) | 76,013 (20.0) | <0.001 |
| Pressure ulcers | 251 (0.6) | 18,650 (6.9) | 17,471 (19.9) | 36,372 (9.2) | <0.001 |
| Prior gastrostomy tube | 275 (0.7) | 7,943 (2.8) | 6,924 (7.6) | 15,142 (3.7) | <0.001 |
| Prior tracheostomy | 345 (0.9) | 7,359 (2.6) | 4,879 (5.2) | 12,583 (3.0) | <0.001 |
| Severe sepsis with/without septic shock | 2,103 (5.7) | 84,886 (32.6) | 38,174 (45.1) | 125,163 (32.8) | <0.001 |
| Shock | 7,043 (19.8) | 106,372 (41.5) | 37,523 (45.1) | 150,938 (40.2) | <0.001 |
| **Primary admission diagnosis category** |  |  |  |  |  |
| Certain infectious and parasitic diseases | 2,695 (7.5) | 78,707 (30.2) | 35,061 (41.0) | 116,463 (30.5) | <0.001 |
| Neoplasms | 1,848 (5.3) | 7,608 (3.0) | 1,162 (1.5) | 10,618 (2.8) |  |
| Diseases of the blood and blood-forming organs, and certain disorders involving the immune mechanism | 163 (0.5) | 1,060 (0.4) | 244 (0.3) | 1,467 (0.4) |  |
| Endocrine, nutritional, and metabolic diseases | 221 (0.6) | 2,207 (0.9) | 749 (0.9) | 3,177 (0.8) |  |
| Diseases of the nervous system | 510 (1.4) | 5,108 (2.0) | 2,525 (3.1) | 8,143 (2.2) |  |
| Diseases of the circulatory system | 14,998 (42.8) | 60,509 (24.2) | 15,713 (19.4) | 91,220 (24.9) |  |
| Diseases of the respiratory system | 8,029 (23.1) | 50,191 (20.1) | 10,072 (12.4) | 68,292 (18.7) |  |
| Diseases of the digestive system | 2,935 (8.3) | 16,326 (6.4) | 3,169 (4.0) | 22,430 (6.1) |  |
| Diseases of the genitourinary system | 168 (0.5) | 3,819 (1.5) | 1,846 (2.3) | 5,833 (1.6) |  |
| Injury, poisoning, and certain other consequences of external causes | 2,485 (7.1) | 22,005 (8.8) | 10,143 (12.7) | 34,633 (9.5) |  |
| Other | 1,074 (3.1) | 6,171 (2.5) | 1,889 (2.3) | 9,134 (2.5) |  |
| **Interventions/treatments** |  |  |  |  |  |
| Renal replacement therapy | 724 (2.0) | 23,142 (9.0) | 7,507 (9.0) | 31,373 (8.3) | <0.001 |
| Insertion of new tracheostomy | 773 (2.2) | 12,816 (5.0) | 6,657 (7.8) | 20,246 (5.3) | <0.001 |
| Insertion of new gastrostomy tube | 616 (1.8) | 13,226 (5.1) | 9,147 (11.0) | 22,989 (6.1) | <0.001 |
| Referral to palliative care | 6,926 (19.9) | 66,890 (27.1) | 22,642 (28.8) | 96,458 (26.8) | <0.001 |
| Duration of mechanical ventilation |  |  |  |  | <0.001 |
| <24 hours | 16,658 (47.7) | 68,494 (27.3) | 13,632 (16.9) | 98,784 (26.9) |  |
| 24-96 hours | 11,960 (33.9) | 101,752 (40.4) | 31,690 (39.1) | 145,402 (39.5) |  |
| >96 hours | 3,564 (10.0) | 73,805 (28.5) | 35,348 (41.7) | 112,717 (29.7) |  |
| Unknown | 2,944 (8.5) | 9,660 (3.8) | 1,903 (2.3) | 14,507 (3.9) |  |
| **Outcomes** |  |  |  |  |  |
| Hospital disposition^c^ |  |  |  |  | <0.001 |
| Routine | 5,304 (28.7) | 15,570 (11.9) | 2,967 (5.9) | 23,841 (12.0) |  |
| Home health care | 4,978 (26.0) | 27,433 (20.2) | 8,410 (16.6) | 40,821 (19.9) |  |
| Transfer to skilled nursing facility, intermediate care facility, or other type of facility | 7,649 (40.8) | 85,726 (64.3) | 37,675 (74.5) | 131,050 (64.6) |  |
| Transfer to short-term hospital | 725 (3.9) | 4,568 (3.4) | 1,440 (2.8) | 6,733 (3.3) |  |
| Discharge alive, but destination unknown | 119 (0.7) | 300 (0.3) | 118 (0.3) | 537 (0.3) |  |
| 30-day in-hospital mortality^d^ | 16,431 (46.7) | 118,008 (46.6) | 30,104 (36.6) | 164,543 (44.4) | <0.001 |
| Hospital costs, median USD (IQR)^e^ | 16,809 (8,003-32,193) | 27,200 (14,573-49,769) | 37,323 (21,662-64,322) | 28,212 (14,947-51,521) | <0.001 |
| **30-day readmission** |  |  |  |  |  |
| Primary admission diagnosis category for readmission^f^ |  |  |  |  | <0.001 |
| Certain infectious and parasitic diseases | 440 (13.9) | 6,762 (23.0) | 3,647 (31.7) | 10,849 (24.6) |  |
| Neoplasms | 64 (2.0) | 327 (1.1) | 88 (0.8) | 479 (1.1) |  |
| Diseases of the blood and blood-forming organs, and certain disorders involving the immune mechanism | 40 (1.4) | 463 (1.6) | 175 (1.6) | 678 (1.6) |  |
| Endocrine, nutritional, and metabolic diseases | 59 (1.9) | 657 (2.4) | 283 (2.7) | 999 (2.4) |  |
| Diseases of the nervous system | 64 (2.0) | 702 (2.5) | 380 (3.7) | 1,146 (2.8) |  |
| Diseases of the circulatory system | 929 (29.8) | 6,218 (22.8) | 1,670 (15.9) | 8,817 (21.6) |  |
| Diseases of the respiratory system | 677 (21.7) | 5,835 (21.0) | 1,776 (16.4) | 8,288 (19.9) |  |
| Diseases of the digestive system | 325 (10.6) | 2,569 (9.3) | 835 (7.8) | 3,729 (9.0) |  |
| Diseases of the genitourinary system | 122 (3.9) | 1,490 (5.4) | 712 (6.7) | 2,324 (5.6) |  |
| Injury, poisoning, and certain other consequences of external causes | 243 (7.7) | 1,970 (6.9) | 885 (8.5) | 3,098 (7.4) |  |
| Other | 164 (5.1) | 1,108 (4.0) | 427 (4.2) | 1,699 (4.1) |  |
| Mechanical ventilation on 30-day readmission^f^ | 535 (17.4) | 7,369 (24.8) | 3,554 (30.4) | 11,458 (25.7) | <0.001 |
| Palliative care referral on 30-day readmission^f^ | 1,859 (13.3) | 1,992 (14.3) | 2,006 (14.6) | 5,857 (14.1) | 0.04 |
| In-hospital mortality for 30-day readmission^f^ | 288 (9.2) | 3,841 (13.3) | 1,602 (14.2) | 5,731 (13.2) | <0.001 |

Abbreviations: acute decompensated heart failure (ADHF), acute kidney injury (AKI), acute respiratory distress syndrome (ARDS), chronic kidney disease (CKD), interquartile range (IQR), United States dollars (USD)

*The Nationwide Readmissions Database (NRD) and Healthcare Cost and Utilization Project (HCUP) discourages the publication of cell counts ≤10

^a^Expressed as unweighted number and weighted percentage (%) unless otherwise stated. Weighted percentages are calculated using complex survey methods in Stata and use the weighted number of hospitalizations.

^b^A p-value <0.05 considered statistically significant.

^c^Among patient hospitalizations that survived their index admission (Unweighted total *n*=18,775 for low-risk, *n*=133,597 for intermediate-risk, *n*=50,610 for high-risk, *n=*202,982 total)

^d^30-day hospital mortality was determined from inpatient hospitalization data and did not include out-of-hospital deaths.

^e^Hospital costs inflation adjusted to 2018 United States dollars

^f^Among patient hospitalizations that were readmitted to hospital at 30-days (Unweighted total *n*=3,127 for low-risk, *n*=28,101 for intermediate-risk, *n*=10,878 for high-risk, *n=*42,106 total)

### eTable 5. Key variables with missing data.

| **Variable** | **Number (%) missing^a^**  *n=*371,410 |
| --- | --- |
| In-hospital mortality | 198 (0.05) |
| Elective admission | 450 (0.12) |
| Income quartile by patient ZIP code | 4,170 (1.12) |
| Hospital disposition | 198 (0.05) |
| Insurance status | 364 (0.10) |
| Hospital costs | 3,360 (0.90) |
| Time to receipt of mechanical ventilation | 6,071 (1.63) |
| Duration of mechanical ventilation | 14,507 (3.90) |

^a^Weighted percentage calculated.

### eTable 6. Model performance of HFRS as a continuous variable and outcome in older adults receiving mechanical ventilation

| **Outcome** | **Unadjusted analysis** | **Adjusted analysis^a^** |
| --- | --- | --- |
| **In-hospital mortality** | | |
| No. of unweighted hospitalizations in analysis | 371,212 | 366,684 |
| HFRS (continuous), OR (95% CI)^b^ | 0.97 (0.97-0.98) | 0.95 (0.94-0.95) |
| C-statistic of the model | 0.533 (0.531-0.535) | 0.714 (0.712-0.716) |
| Brier score of the model | 0.247 | 0.214 |
| **Prolonged hospital length of stay (>10 days)** | | |
| No. of unweighted hospitalizations in analysis | 371,410 | 366,881 |
| HFRS (continuous), OR (95% CI)^b^ | 1.11 (1.11-1.12) | 1.11 (1.11-1.11) |
| C-statistic of the model | 0.651 (0.649-0.653) | 0.707 (0.705-0.708) |
| Brier score of the model | 0.227 | 0.213 |
| **30-day readmission** | | |
| No. of unweighted hospitalizations in analysis^c^ | 202,982 | 200,006 |
| HFRS (continuous), OR (95% CI)^b^ | 1.01 (1.01-1.01) | 1.01 (1.00-1.01) |
| C-statistic of model | 0.517 (0.514-0.520) | 0.595 (0.952-0.598) |
| Brier score of model | 0.164 | 0.162 |

Abbreviations: confidence interval (CI), hospital frailty risk score (HFRS), number (No.), odds ratio (OR)

^a^Adjusted for age (continuous variable), Elixhauser-van Walraven comorbidity index score (continuous variable), do-not-resuscitate status, biological sex, insurance status, income quartile, year of study, hospital teaching status, hospital size, and admission diagnosis category. 30-day emergency readmissions include adjustment for all prior variables and additionally for hospital disposition.

^b^For each 1-unit increase in HFRS, for continuous model

^c^Total number of patient hospitalizations in analysis who survived index hospital admission.

### eTable 7. Model performance of HFRS using restricted cubic splines and outcome in older adults receiving mechanical ventilation

| **Outcome** | **Unadjusted analysis** | **Adjusted analysis^a^** |
| --- | --- | --- |
| **In-hospital mortality** | | |
| No. of unweighted hospitalizations in analysis | 371,212 | 366,684 |
| HFRS^b^, OR |  |  |
| 0 | 1.40 (1.24-1.57) | 1.84 (1.64-2.06) |
| 5 | 1.00 (Reference) | 1.00 (Reference) |
| 10 | 1.14 (1.11-1.16) | 0.91 (0.89-0.94) |
| 15 | 0.95 (0.92-0.97) | 0.67 (0.66-0.69) |
| C-statistic of the model | 0.541 (0.539-0.543) | 0.715 (0.713-0.716) |
| Brier score of the model | 0.246 | 0.214 |
| **Prolonged hospital length of stay (>10 days)** | | |
| No. of unweighted hospitalizations in analysis | 371,410 | 366,881 |
| HFRS^b^, OR |  |  |
| 0 | 0.45 (0.32-0.63) | 0.49 (0.35-0.69) |
| 5 | 1.00 (Reference) | 1.00 (Reference) |
| 10 | 2.34 (2.26-2.41) | 2.17 (2.10-2.24) |
| 15 | 3.69 (3.57-3.81) | 3.49 (3.38-3.61) |
| C-statistic of the model | 0.650 (0.649-0.652) | 0.707 (0.705-0.709) |
| Brier score of the model | 0.226 | 0.212 |
| **30-day readmission** | | |
| No. of unweighted hospitalizations in analysis^c^ | 202,982 | 200,006 |
| HFRS^b^, OR |  |  |
| 0 | 0.55 (0.47-0.65) | 0.65 (0.55-0.77) |
| 5 | 1.00 (Reference) | 1.00 (Reference) |
| 10 | 1.15 (1.11-1.19) | 1.07 (1.03-1.12) |
| 15 | 1.16 (1.12-1.21) | 1.10 (1.05-1.14) |
| C-statistic of model | 0.519 (0.516-0.522) | 0.596 (0.592-0.599) |
| Brier score of model | 0.164 | 0.162 |

Abbreviations: confidence interval (CI), hospital frailty risk score (HFRS), number (No.), odds ratio (OR)

^a^Adjusted for age (continuous variable), Elixhauser-van Walraven comorbidity index score (continuous variable), do-not-resuscitate status, biological sex, insurance status, income quartile, year of study, hospital teaching status, hospital size, and admission diagnosis category. 30-day emergency readmissions include adjustment for all prior variables and additionally for hospital disposition.

^b^HFRS as continuous variable using restricted cubic splines with an HFRS of 5 as the reference category

^b^Total number of patient hospitalizations in analysis who survived index hospital admission.

### eTable 8. Hospital frailty risk score subcategory and adverse outcomes in older adults receiving mechanical ventilation, using a Cox proportional hazards model

| **Outcome** | **Adjusted analysis^a^** |
| --- | --- |
| **In-hospital mortality** |  |
| No. of unweighted hospitalizations in analysis | 350,749 |
| Low-risk HFRS, adjusted HR (95% CI) | 1.00 (Reference) |
| Intermediate-risk HFRS, adjusted HR (95% CI) | 0.67 (0.66-0.69) |
| High-risk HFRS, adjusted HR (95% CI) | 0.40 (0.39-0.41) |
| **30-day hospital readmission** |  |
| No. of unweighted hospitalizations in analysis^b^ | 200,540 |
| Low-risk HFRS, adjusted HR (95% CI) | 1.00 (Reference) |
| Intermediate-risk HFRS, adjusted HR (95% CI) | 1.16 (1.11-1.21) |
| High-risk HFRS, adjusted HR (95% CI) | 1.18 (1.12-1.24) |

Abbreviations: confidence interval (CI), hospital frailty risk score (HFRS), hazard ratio (HR), number (No.)

^a^Adjusted for age (continuous variable), Elixhauser-van Walraven comorbidity index score (continuous variable), do-not-resuscitate status, biological sex, insurance status, income quartile, year of study, hospital teaching status, hospital size, and admission diagnosis category. 30-day emergency readmissions include adjustment for all prior variables and additionally for hospital disposition.

^b^Total number of patient hospitalizations in analysis who survived index hospital admission.

### eTable 9. Model performance of HFRS subcategory and in-hospital 30-day mortality in older adults receiving mechanical ventilation

| **Outcome** | **Unadjusted analysis** | **Adjusted analysis^a^** |
| --- | --- | --- |
| **30-day in-hospital mortality** | | |
| No. of unweighted hospitalizations in analysis | 371,206 | 366,678 |
| Low-risk HFRS, OR (95% CI) | 1.00 (Reference) | 1.00 (Reference) |
| Intermediate-risk HFRS, OR (95% CI) | 0.99 (0.97-1.02) | 0.78 (0.76-0.80) |
| High-risk HFRS, OR (95% CI) | 0.66 (0.64-0.68) | 0.43 (0.42-0.45) |
| C-statistic of the model | 0.535 (0.533-0.537) | 0.709 (0.708-0.711) |
| Brier score of the model | 0.245 | 0.214 |

Abbreviations: confidence interval (CI), hospital frailty risk score (HFRS), number (No.), odds ratio (OR)

^a^Adjusted for age (continuous variable), Elixhauser-van Walraven comorbidity index score (continuous variable), do-not-resuscitate status, biological sex, insurance status, income quartile, year of study, hospital teaching status, hospital size, and admission diagnosis category.

### eTable 10. Model performance of HFRS subcategory and outcome in older adult mechanically ventilated patients who received mechanical ventilation for greater than 24 hours

| **Outcome** | **Unadjusted analysis** | **Adjusted analysis^a^** |
| --- | --- | --- |
| **In-hospital mortality** | | |
| No. of unweighted hospitalizations in analysis | 250,858 | 247,897 |
| Low-risk HFRS, OR (95% CI) | 1.00 (Reference) | 1.00 (Reference) |
| Intermediate-risk HFRS, OR (95% CI) | 1.49 (1.42-1.55) | 1.06 (1.00-1.12) |
| High-risk HFRS, OR (95% CI) | 1.17 (1.11-1.22) | 0.70 (0.65-0.74) |
| C-statistic of the model | 0.530 (0.528-0.532) | 0.724 (0.722-0.726) |
| Brier score of the model | 0.236 | 0.202 |
| **Prolonged hospital length of stay (>10 days)** | | |
| No. of unweighted hospitalizations in analysis | 250,996 | 248,034 |
| Low-risk HFRS, OR (95% CI) | 1.00 (Reference) | 1.00 (Reference) |
| Intermediate-risk HFRS, OR (95% CI) | 2.24 (2.10-2.39) | 2.02 (1.89-2.15) |
| High-risk HFRS, OR (95% CI) | 4.04 (3.77-4.32) | 3.65 (3.40-3.92) |
| C-statistic of the model | 0.577 (0.575-0.579) | 0.667 (0.664-0.669) |
| Brier score of the model | 0.243 | 0.229 |
| **30-day readmission** | | |
| No. of unweighted hospitalizations in analysis^b^ | 153,829 | 151,660 |
| Low-risk HFRS, OR (95% CI) | 1.00 (Reference) | 1.00 (Reference) |
| Intermediate-risk HFRS, OR (95% CI) | 1.24 (1.16-1.32) | 1.15 (1.08-1.22) |
| High-risk HFRS, OR (95% CI) | 1.24 (1.16-1.33) | 1.17 (1.09-1.26) |
| C-statistic of model | 0.513 (0.510-0.516) | 0.590 (0.586-0.593) |
| Brier score of model | 0.164 | 0.166 |

Abbreviations: confidence interval (CI), hospital frailty risk score (HFRS), number (No.), odds ratio (OR)

^a^Adjusted for age (continuous variable), Elixhauser-van Walraven comorbidity index score (continuous variable), do-not-resuscitate status, biological sex, insurance status, income quartile, year of study, hospital teaching status, hospital size, and admission diagnosis category. 30-day emergency readmissions include adjustment for all prior variables and additionally for hospital disposition.

^b^Total number of patient hospitalizations in analysis who survived index hospital admission.

### eTable 11. Model performance of HFRS subcategory and outcome in older mechanically ventilated adults who only were admitted to hospital for an emergency admission

| **Outcome** | **Unadjusted analysis** | **Adjusted analysis^a^** |
| --- | --- | --- |
| **In-hospital mortality** | | |
| No. of unweighted hospitalizations in analysis | 347,679 | 343,489 |
| Low-risk HFRS, OR (95% CI) | 1.00 (Reference) | 1.00 (Reference) |
| Intermediate-risk HFRS, OR (95% CI) | 0.92 (0.89-0.95) | 0.72 (0.70-0.75) |
| High-risk HFRS, OR (95% CI) | 0.63 (0.61-0.66) | 0.42 (0.41-0.44) |
| C-statistic of the model | 0.536 (0.534-0.537) | 0.710 (0.709-0.712) |
| Brier score of the model | 0.247 | 0.216 |
| **Prolonged hospital length of stay (>10 days)** | | |
| No. of unweighted hospitalizations in analysis | 347,867 | 343,676 |
| Low-risk HFRS, OR (95% CI) | 1.00 (Reference) | 1.00 (Reference) |
| Intermediate-risk HFRS, OR (95% CI) | 3.12 (2.93-3.32) | 2.53 (2.37-2.69) |
| High-risk HFRS, OR (95% CI) | 6.77 (6.34-7.23) | 5.40 (5.05-5.78) |
| C-statistic of the model | 0.606 (0.604-0.607) | 0.691 (0.689-0.693) |
| Brier score of the model | 0.230 | 0.216 |
| **30-day readmission** | | |
| No. of unweighted hospitalizations in analysis^b^ | 187,521 | 184,773 |
| Low-risk HFRS, OR (95% CI) | 1.00 (Reference) | 1.00 (Reference) |
| Intermediate-risk HFRS, OR (95% CI) | 1.27 (1.20-1.33) | 1.14 (1.08-1.20) |
| High-risk HFRS, OR (95% CI) | 1.29 (1.22-1.37) | 1.16 (1.09-1.23) |
| C-statistic of model | 0.513 (0.510-0.516) | 0.595 (0.592-0.598) |
| Brier score of model | 0.164 | 0.162 |

Abbreviations: confidence interval (CI), hospital frailty risk score (HFRS), number (No.), odds ratio (OR)

^a^Adjusted for age (continuous variable), Elixhauser-van Walraven comorbidity index score (continuous variable), do-not-resuscitate status, biological sex, insurance status, income quartile, year of study, hospital teaching status, hospital size, and admission diagnosis category. 30-day emergency readmissions include adjustment for all prior variables and additionally for hospital disposition.

^b^Total number of patient hospitalizations in analysis who survived index hospital admission.

### eTable 12. Model performance of HFRS subcategory and outcome in older mechanically ventilated adults who only were admitted to hospital and had a major operative procedure

| **Outcome** | **Unadjusted analysis** | **Adjusted analysis^a^** |
| --- | --- | --- |
| **In-hospital mortality** | | |
| No. of unweighted hospitalizations in analysis | 61,392 | 60,667 |
| Low-risk HFRS, OR (95% CI) | 1.00 (Reference) | 1.00 (Reference) |
| Intermediate-risk HFRS, OR (95% CI) | 1.46 (1.36-1.57) | 0.98 (0.90-1.06) |
| High-risk HFRS, OR (95% CI) | 1.06 (0.98-1.15) | 0.58 (0.53-0.64) |
| C-statistic of the model | 0.537 (0.533-0.541) | 0.717 (0.713-0.722) |
| Brier score of the model | 0.227 | 0.194 |
| **Prolonged hospital length of stay (>10 days)** | | |
| No. of unweighted hospitalizations in analysis | 61,422 | 60,697 |
| Low-risk HFRS, OR (95% CI) | 1.00 (Reference) | 1.00 (Reference) |
| Intermediate-risk HFRS, OR (95% CI) | 3.52 (3.28-3.77) | 2.97 (2.76-3.19) |
| High-risk HFRS, OR (95% CI) | 7.70 (7.09-8.37) | 6.59 (6.01-7.22) |
| C-statistic of the model | 0.619 (0.615-0.623) | 0.713 (0.709-0.718) |
| Brier score of the model | 0.220 | 0.203 |
| **30-day readmission** | | |
| No. of unweighted hospitalizations in analysis^b^ | 39,765 | 39,246 |
| Low-risk HFRS, OR (95% CI) | 1.00 (Reference) | 1.00 (Reference) |
| Intermediate-risk HFRS, OR (95% CI) | 1.35 (1.23-1.48) | 1.22 (1.10-1.34) |
| High-risk HFRS, OR (95% CI) | 1.34 (1.20-1.49) | 1.19 (1.06-1.34) |
| C-statistic of model | 0.509 (0.507-0.512) | 0.590 (0.587-0.594) |
| Brier score of model | 0.164 | 0.162 |

Abbreviations: confidence interval (CI), hospital frailty risk score (HFRS), number (No.), odds ratio (OR)

^a^Adjusted for age (continuous variable), Elixhauser-van Walraven comorbidity index score (continuous variable), do-not-resuscitate status, biological sex, insurance status, income quartile, year of study, hospital teaching status, hospital size, and admission diagnosis category. 30-day emergency readmissions include adjustment for all prior variables and additionally for hospital disposition.

^b^Total number of patient hospitalizations in analysis who survived index hospital admission.

### eTable 13. Model performance of HFRS subcategory and outcome in older mechanically ventilated adults who only were admitted to hospital and did not have a major operative procedure

| **Outcome** | **Unadjusted analysis** | **Adjusted analysis^a^** |
| --- | --- | --- |
| **In-hospital mortality** | | |
| No. of unweighted hospitalizations in analysis | 309,820 | 306,017 |
| Low-risk HFRS, OR (95% CI) | 1.00 (Reference) | 1.00 (Reference) |
| Intermediate-risk HFRS, OR (95% CI) | 0.94 (0.91-0.97) | 0.75 (0.73-0.78) |
| High-risk HFRS, OR (95% CI) | 0.65 (0.63-0.68) | 0.44 (0.42-0.45) |
| C-statistic of the model | 0.534 (0.533-0.536) | 0.711 (0.709-0.713) |
| Brier score of the model | 0.248 | 0.216 |
| **Prolonged hospital length of stay (>10 days)** | | |
| No. of unweighted hospitalizations in analysis | 309,988 | 306,184 |
| Low-risk HFRS, OR (95% CI) | 1.00 (Reference) | 1.00 (Reference) |
| Intermediate-risk HFRS, OR (95% CI) | 3.35 (3.11-3.60) | 2.62 (2.43-2.82) |
| High-risk HFRS, OR (95% CI) | 7.54 (6.99-8.14) | 5.76 (5.33-6.23) |
| C-statistic of the model | 0.611 (0.610-0.613) | 0.691 (0.689-0.693) |
| Brier score of the model | 0.222 | 0.210 |
| **30-day readmission** | | |
| No. of unweighted hospitalizations in analysis^b^ | 163,217 | 160,760 |
| Low-risk HFRS, OR (95% CI) | 1.00 (Reference) | 1.00 (Reference) |
| Intermediate-risk HFRS, OR (95% CI) | 1.31 (1.24-1.38) | 1.16 (1.0-1.23) |
| High-risk HFRS, OR (95% CI) | 1.34 (1.27-1.42) | 1.19 (1.12-1.27) |
| C-statistic of model | 0.513 (0.510-0.516) | 0.595 (0.592-0.598) |
| Brier score of model | 0.164 | 0.162 |

Abbreviations: confidence interval (CI), hospital frailty risk score (HFRS), number (No.), odds ratio (OR)

^a^Adjusted for age (continuous variable), Elixhauser-van Walraven comorbidity index score (continuous variable), do-not-resuscitate status, biological sex, insurance status, income quartile, year of study, hospital teaching status, hospital size, and admission diagnosis category. 30-day emergency readmissions include adjustment for all prior variables and additionally for hospital disposition.

^b^Total number of patient hospitalizations in analysis who survived index hospital admission.

### eTable 14. Model performance of HFRS subcategory and outcome in older mechanically ventilated adults who only were admitted to hospital, after additional adjustment for time to receipt of mechanical ventilation

| **Outcome** | **Adjusted analysis^a^** |
| --- | --- |
| **In-hospital mortality** |  |
| No. of unweighted hospitalizations in analysis | 360,686 |
| Low-risk HFRS, OR (95% CI) | 1.00 (Reference) |
| Intermediate-risk HFRS, OR (95% CI) | 0.77 (0.74-0.79) |
| High-risk HFRS, OR (95% CI) | 0.43 (0.42-0.45) |
| C-statistic of the model | 0.720 (0.718-0.722) |
| Brier score of the model | 0.213 |
| **Prolonged hospital length of stay (>10 days)** |  |
| No. of unweighted hospitalizations in analysis | 360,883 |
| Low-risk HFRS, OR (95% CI) | 1.00 (Reference) |
| Intermediate-risk HFRS, OR (95% CI) | 2.40 (2.28-2.53) |
| High-risk HFRS, OR (95% CI) | 5.03 (4.76-5.32) |
| C-statistic of the model | 0.760 (0.759-0.762) |
| Brier score of the model | 0.192 |
| **30-day readmission** |  |
| No. of unweighted hospitalizations in analysis^b^ | 196,283 |
| Low-risk HFRS, OR (95% CI) | 1.00 (Reference) |
| Intermediate-risk HFRS, OR (95% CI) | 1.18 (1.12-1.24) |
| High-risk HFRS, OR (95% CI) | 1.21 (1.14-1.28) |
| C-statistic of model | 0.597 (0.594-0.600) |
| Brier score of model | 0.163 |

Abbreviations: confidence interval (CI), hospital frailty risk score (HFRS), number (No.), odds ratio (OR)

^a^Adjusted for time to receipt of mechanical ventilation, age (continuous variable), Elixhauser-van Walraven comorbidity index score (continuous variable), do-not-resuscitate status, biological sex, insurance status, income quartile, year of study, hospital teaching status, hospital size, and admission diagnosis category. 30-day emergency readmissions include adjustment for all prior variables and additionally for hospital disposition.

^b^Total number of patient hospitalizations in analysis who survived index hospital admission.

### eTable 15. Model performance of HFRS subcategory and outcome in older adults receiving mechanical ventilation after multiple imputation with chained equations

| **Outcome** | **Unadjusted analysis** | **Adjusted analysis^a^** |
| --- | --- | --- |
| **In-hospital mortality** | | |
| No. of unweighted hospitalizations in analysis | 371,410 | 371,410 |
| Low-risk HFRS, OR (95% CI) | 1.00 (Reference) | 1.00 (Reference) |
| Intermediate-risk HFRS, OR (95% CI) | 1.03 (1.00-1.07) | 0.79 (0.77-0.82) |
| High-risk HFRS, OR (95% CI) | 0.73 (0.70-0.75) | 0.46 (0.45-0.48) |
| C-statistic of the model | 0.548 (0.548-0.549) | 0.762 (0.761-0.762) |
| Brier score of the model | 0.218 | 0.180 |
| **Prolonged hospital length of stay (>10 days)** | | |
| No. of unweighted hospitalizations in analysis | 371,410 | 371,410 |
| Low-risk HFRS, OR (95% CI) | 1.00 (Reference) | 1.00 (Reference) |
| Intermediate-risk HFRS, OR (95% CI) | 3.11 (2.93-3.29) | 2.61 (2.46-2.77) |
| High-risk HFRS, OR (95% CI) | 6.68 (6.27-7.10) | 5.60 (5.25-5.97) |
| C-statistic of the model | 0.679 (0.678-0.679) | 0.723 (0.722-0.723) |
| Brier score of the model | 0.142 | 0.130 |
| **30-day readmission** | | |
| No. of unweighted hospitalizations in analysis^b^ | 202,982 | 202,445 |
| Low-risk HFRS, OR (95% CI) | 1.00 (Reference) | 1.00 (Reference) |
| Intermediate-risk HFRS, OR (95% CI) | 1.32 (1.26-1.38) | 1.18 (1.12-1.23) |
| High-risk HFRS, OR (95% CI) | 1.35 (1.27-1.42) | 1.20 (1.13-1.27) |
| C-statistic of model | 0.531 (0.531-0.531) | 0.582 (0.582-0.583) |
| Brier score of model | 0.126 | 0.128 |

Abbreviations: confidence interval (CI), hospital frailty risk score (HFRS), number (No.), odds ratio (OR)

^a^Adjusted for age (continuous variable), Elixhauser-van Walraven comorbidity index score (continuous variable), do-not-resuscitate status, biological sex, insurance status, income quartile, year of study, hospital teaching status, hospital size, and admission diagnosis category. 30-day emergency readmissions include adjustment for all prior variables and additionally for hospital disposition.

^b^Total number of patient hospitalizations in analysis who survived index hospital admission.

### eTable 16. Model performance of HFRS subcategory and outcome in all hospitalized older adults

| **Outcome** | **Unadjusted analysis** | **Adjusted analysis^a^** |
| --- | --- | --- |
| **In-hospital mortality** | | |
| No. of unweighted hospitalizations in analysis | 10,660,304 | 10,531,415 |
| Low-risk HFRS, OR (95% CI) | 1.00 (Reference) | 1.00 (Reference) |
| Intermediate-risk HFRS, OR (95% CI) | 3.92 (3.86-3.99) | 1.92 (1.89-1.95) |
| High-risk HFRS, OR (95% CI) | 6.74 (6.58-6.89) | 2.10 (2.05-2.15) |
| C-statistic of the model | 0.648 (0.648-0.649) | 0.845 (0.845-0.846) |
| Brier score of the model | 0.043 | 0.038 |
| **Prolonged hospital length of stay (>10 days)** | | |
| No. of unweighted hospitalizations in analysis | 10,663,511 | 10,534,562 |
| Low-risk HFRS, OR (95% CI) | 1.00 (Reference) | 1.00 (Reference) |
| Intermediate-risk HFRS, OR (95% CI) | 3.74 (3.66-3.81) | 3.35 (3.28-3.42) |
| High-risk HFRS, OR (95% CI) | 11.12 (10.82-11.43) | 9.51 (9.24-9.78) |
| C-statistic of the model | 0.679 (0.678-0.679) | 0.751 (0.750-0.751) |
| Brier score of the model | 0.090 | 0.086 |
| **30-day readmission** | | |
| No. of unweighted hospitalizations in analysis^b^ | 10,178,153 | 10,055,587 |
| Low-risk HFRS, OR (95% CI) | 1.00 (Reference) | 1.00 (Reference) |
| Intermediate-risk HFRS, OR (95% CI) | 1.39 (1.38-1.39) | 1.15 (1.15-1.16) |
| High-risk HFRS, OR (95% CI) | 1.46 (1.45-1.47) | 1.11 (1.10-1.12) |
| C-statistic of model | 0.539 (0.539-0.540) | 0.613 (0.613-0.614) |
| Brier score of model | 0.128 | 0.126 |

Abbreviations: confidence interval (CI), hospital frailty risk score (HFRS), number (No.), odds ratio (OR)

^a^Adjusted for age (continuous variable), Elixhauser-van Walraven comorbidity index score (continuous variable), do-not-resuscitate status, biological sex, insurance status, income quartile, year of study, hospital teaching status, hospital size, and admission diagnosis category. 30-day emergency readmissions include adjustment for all prior variables and additionally for hospital disposition.

^b^Total number of patient hospitalizations in analysis who survived index hospital admission.

### eFigure 1. Distribution of hospital frailty risk index score


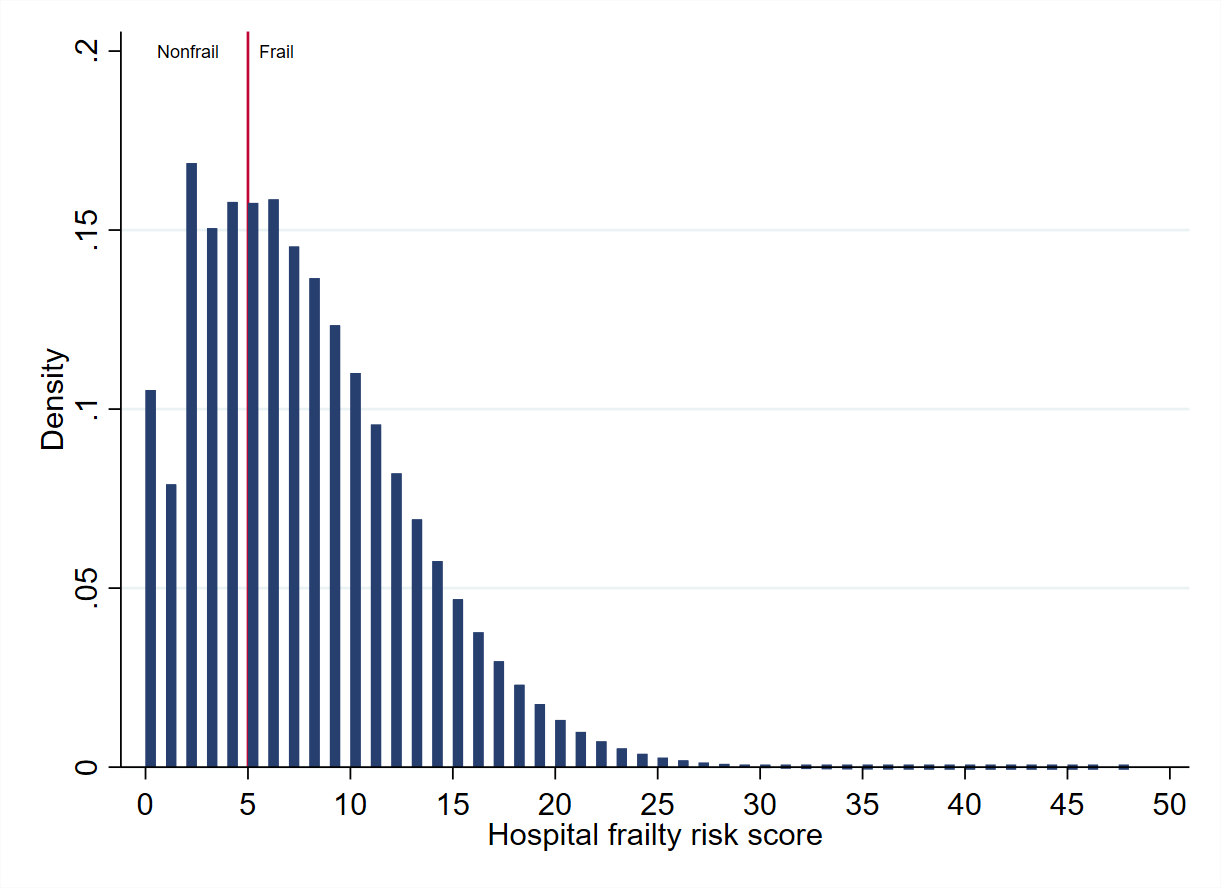


### Electronic Supplementary Material References

1. Gilbert T, Neuburger J, Kraindler J, et al. Development and validation of a Hospital Frailty Risk Score focusing on older people in acute care settings using electronic hospital records: an observational study. *Lancet*. 2018;391(10132):1775-1782. doi:10.1016/S0140-6736(18)30668-8

2. Gilbert T, Cordier Q, Polazzi S, et al. External validation of the Hospital Frailty Risk Score in France. *Age Ageing*. Published online 2021:1-8. doi:10.1093/ageing/afab126

3. Healthcare Cost and Utilization Project (HCUP). Agency for Healthcare Research and Quality (AHRQ). Clinical Classifications Software Refined (CCSR). Published 2020. Accessed December 31, 2020. https://www.hcup-us.ahrq.gov/toolssoftware/ccsr/ccs_refined.jsp
